# Supplementary material for: Incidence of switching to second-line antiretroviral therapy and its predictors among children on antiretroviral therapy at general hospitals, Northern Ethiopia: A survival analysis
Source: PLoS One. 2023 Sep 8;18(9):e0288132. doi: 10.1371/journal.pone.0288132 (PMC10490964; doi:10.1371/journal.pone.0288132)

**Sample of Kaplan-Meier curves showing significant survival difference between categories** (Supporting Figure 1)

As shown below (S1 Fig.), we can note that the survival curves among the categories of each independent variable were not crossed each other. This indicates, there was survival difference between the categories though it could be difficult to figure out the exact figure how much the difference statistically. For instance, children with advanced WHO stage at baseline were more likely to switch to second-line ART regimen compared to children started with early WHO stage **(S1 Figure 1a).** Likewise, children who had Tuberculosis at baseline, suboptimal adherence and anemia after ART initiation had increased chance of switching to second-line ART regimens compared to their counterparts **(S1 Figure 1b, 1c, & 1d).**

**Supporting Figure 1 (S1 Fig.).**

Supporting Figure 1a: Kaplan Meier survival probability plots showing survival differences for baseline WHO stage to assess second-line switching among of HIV/AIDS infected children on first-line ART in public general hospitals, Northern Ethiopia, 2019/20, (n=424)


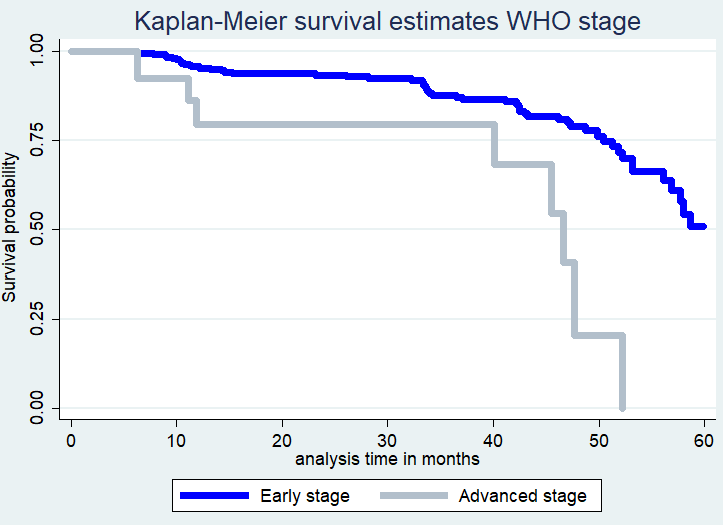


Supporting Figure 1b: Kaplan Meier survival probability plots showing survival differences by tuberculosis at baseline to assess second-line switching among of HIV/AIDS infected children on first-line ART in public general hospitals, Northern Ethiopia, 2019/20, (n=424)


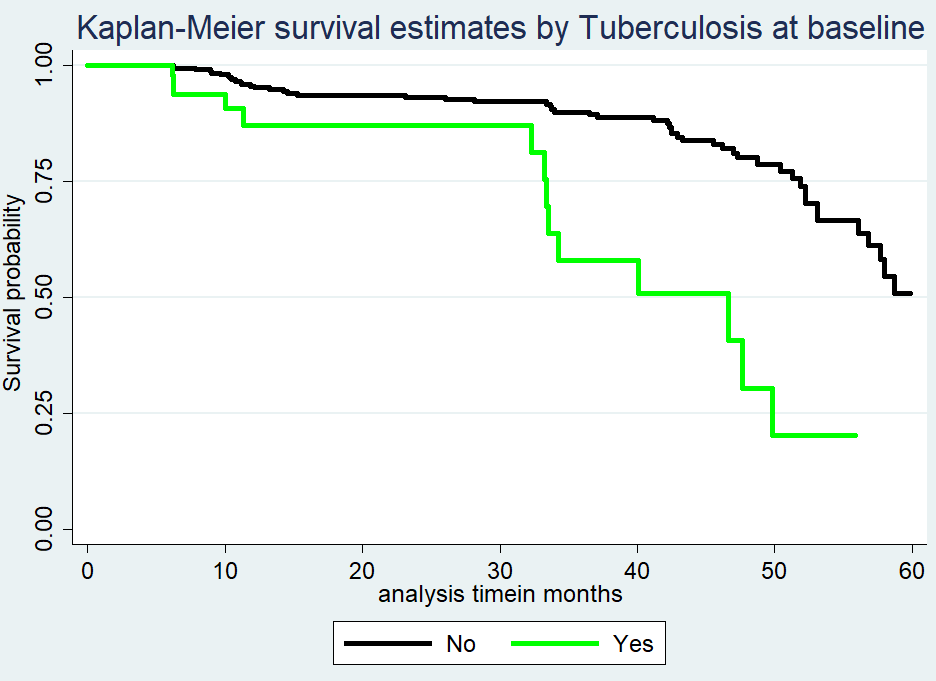


Supporting Figure 1c: Kaplan Meier survival probability plots showing survival differences by anemia after ART initiation to assess second-line switching among of HIV/AIDS infected children on first-line ART in public general hospitals, Northern Ethiopia, 2019/20, (n=424)


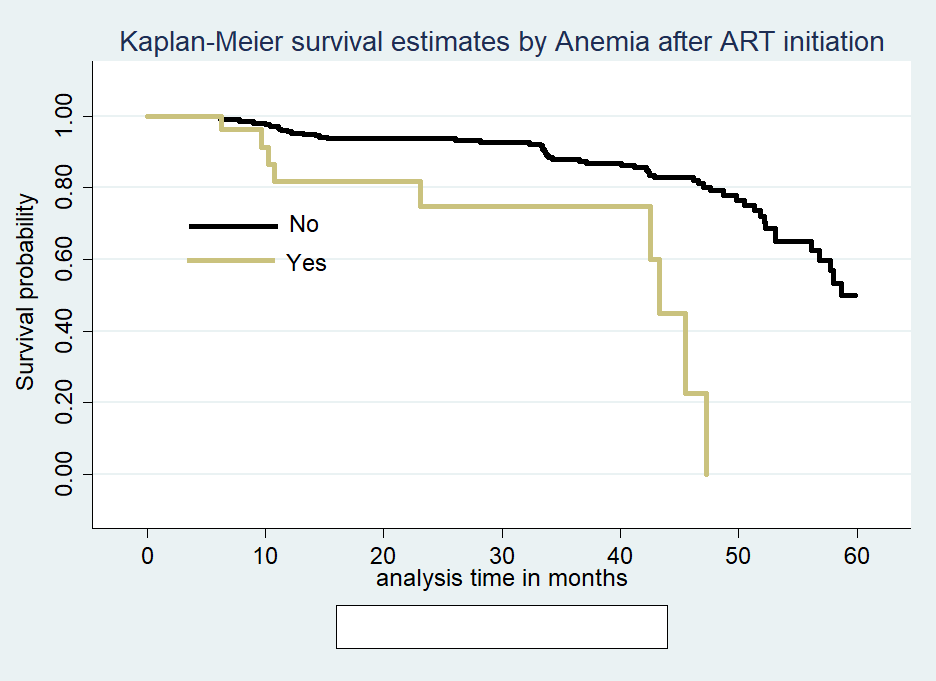


Supporting Figure 1d: Kaplan Meier Survival probability plots showing survival differences by ART drug adherence to assess second-line switching among of HIV/AIDS infected children on first-line ART in public general hospitals, Northern Ethiopia, 2019/20, (n=424)


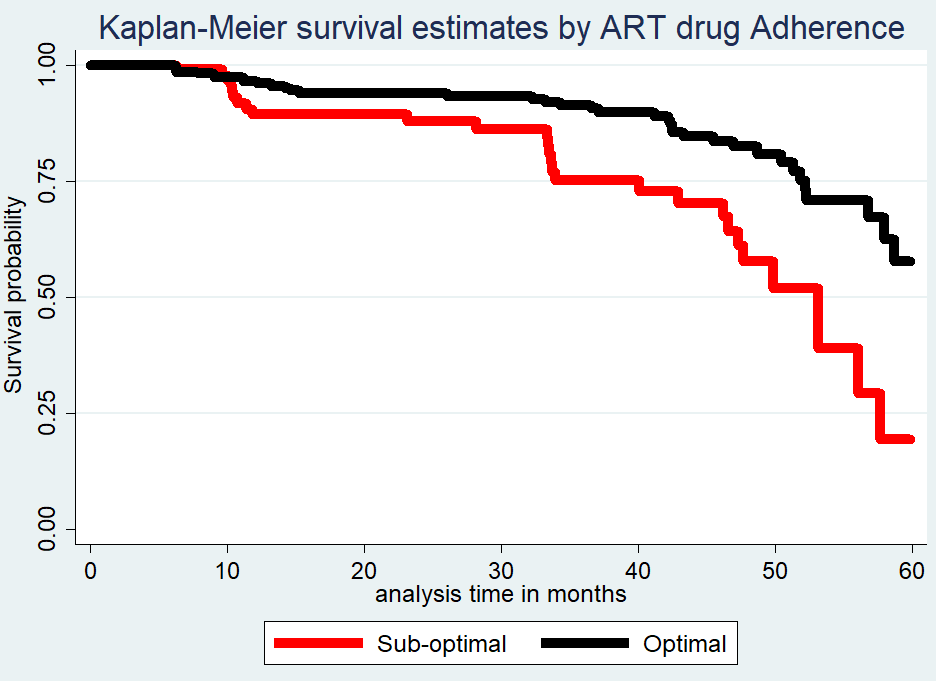


Supporting Figure 1e: Kaplan Meier survival probability plots showing survival differences by ART drug toxicity to assess second-line switching among of HIV/AIDS infected children on first-line ART in public general hospitals, Northern Ethiopia, 2019/20, (n=424)


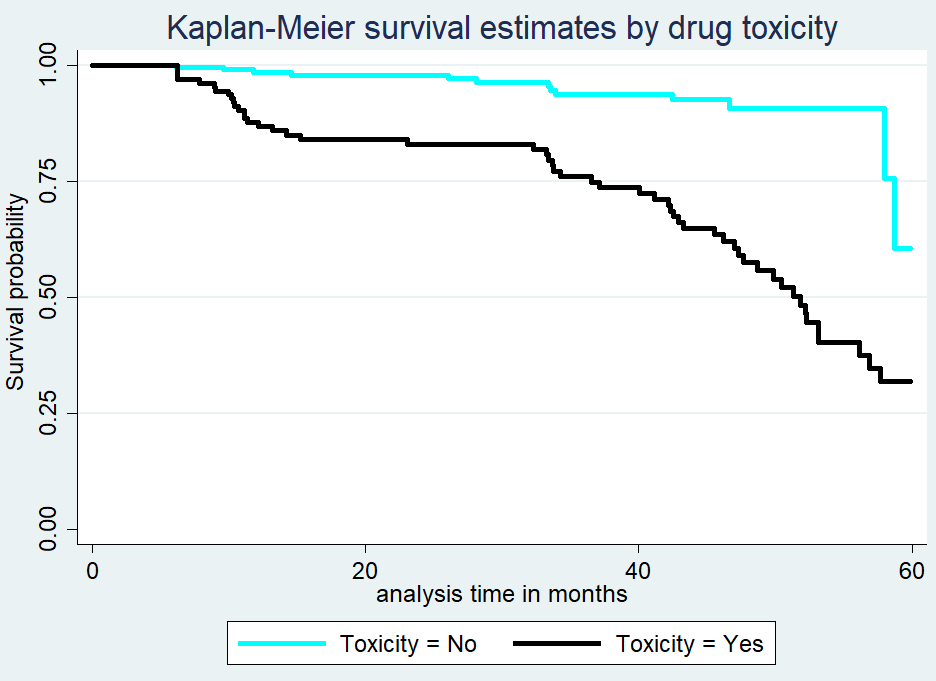


Supporting Figure 1f: Kaplan Meier survival probability plots showing survival differences by opportunistic infections at baseline to assess second-line switching among of HIV/AIDS infected children on first-line ART in public general hospitals, Northern Ethiopia, 2019/20, (n=424)


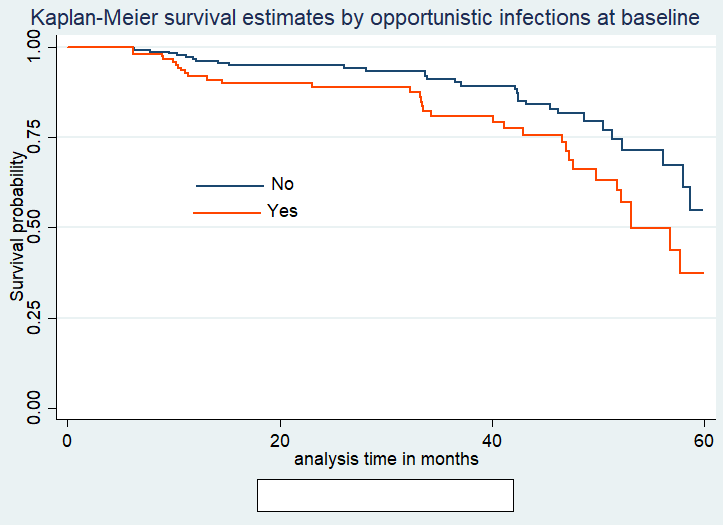


Sample of Kaplan-Meier graphs showing no significant survival difference between categories (Supporting Figure 2)

In the figures below **(**Supporting Figure **2),** we can note that the Kaplan-Meier survival curves among the categories crossed each other, which indicate there was no significant survival difference between each category of the independent variables. For example, the survival curve showed that there is no significant survival difference between being male and female. The same is true for children who received OI prophylaxis and those who did not **(S1 Figure 2a-2d).**

**Supporting Figure 2 (S2 Fig.).**

Supporting Figure 2a: Kaplan-Meier survival probability plots with no survival differences for sex to assess second-line switching among of HIV/AIDS infected children on first-line ART in public general hospitals, Northern Ethiopia, 2019/20, (n=424)


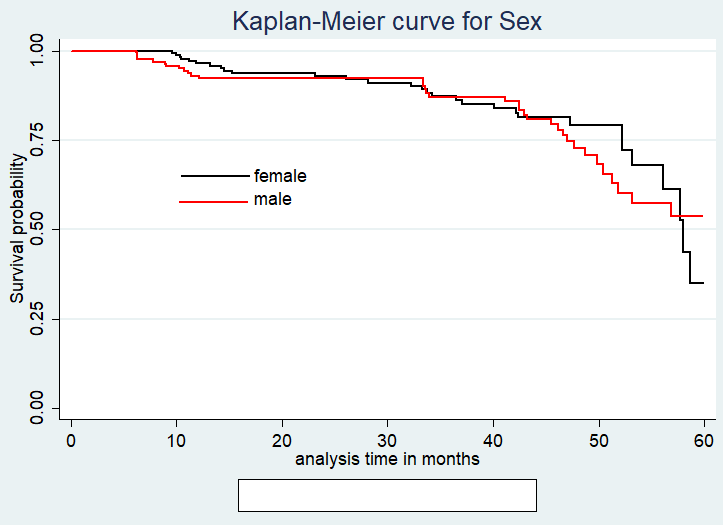


Supporting Figure 2b: Kaplan-Meier survival probability plots with no survival differences for opportunistic infection prophylaxis categories to assess second-line switching among of HIV/AIDS infected children on first-line ART in public general hospitals, Northern Ethiopia, 2019/20, (n=424)


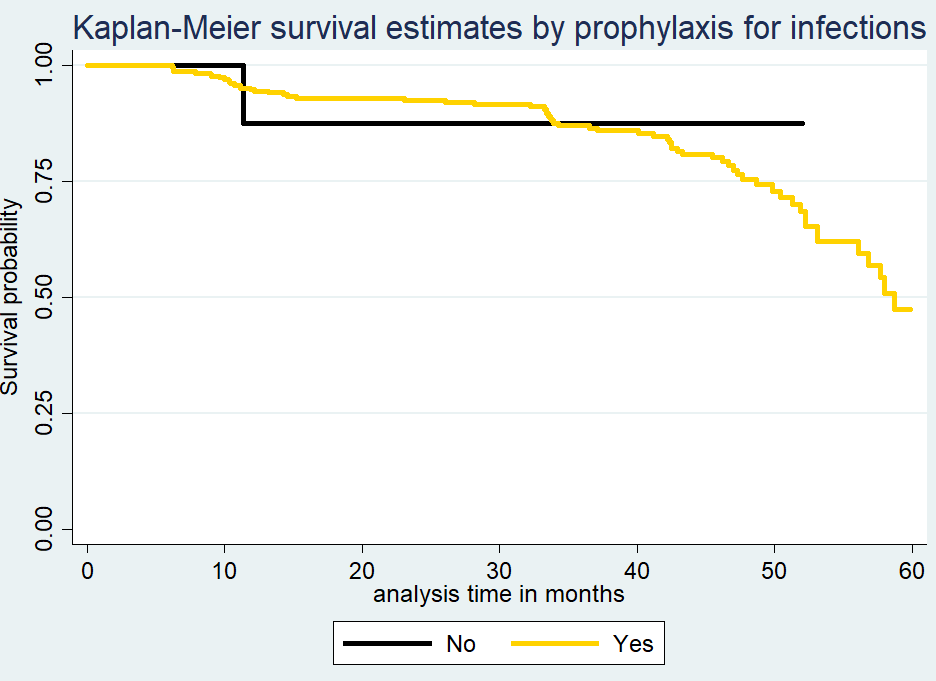


Supporting Figure 2c: Kaplan-Meier survival probability plots with no survival differences for drug regimen change to assess second-line switching among of HIV/AIDS infected children on first-line ART in public general hospitals, Northern Ethiopia, 2019/20, (n=424)


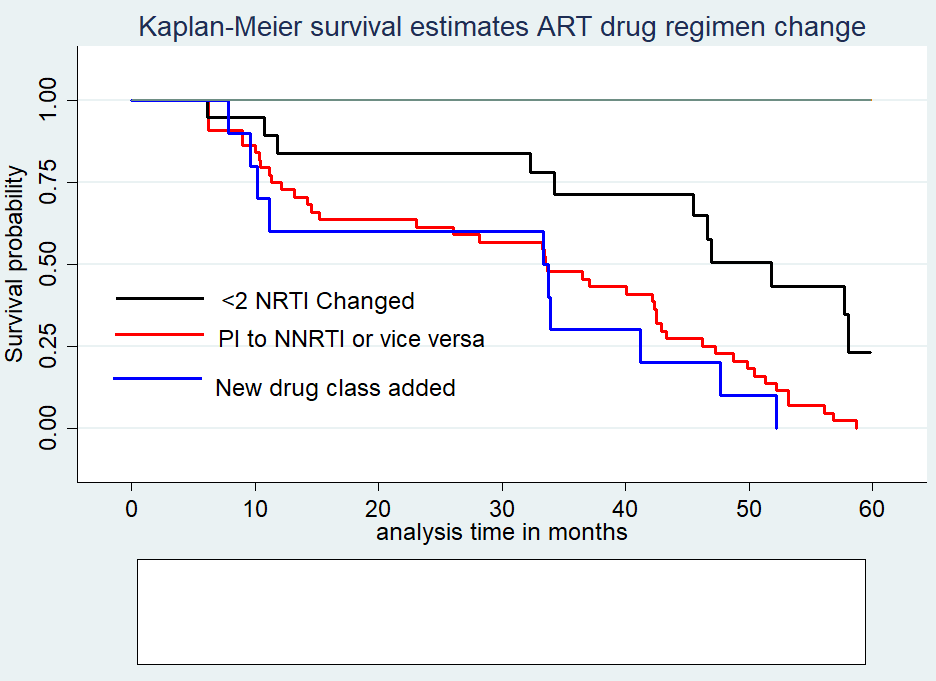


Supporting Figure 2d: Kaplan-Meier survival probability plots with no survival differences ART drug substitution to assess second-line switching among of HIV/AIDS infected children on first-line ART in public general hospitals, Northern Ethiopia, 2019/20, (n=424)


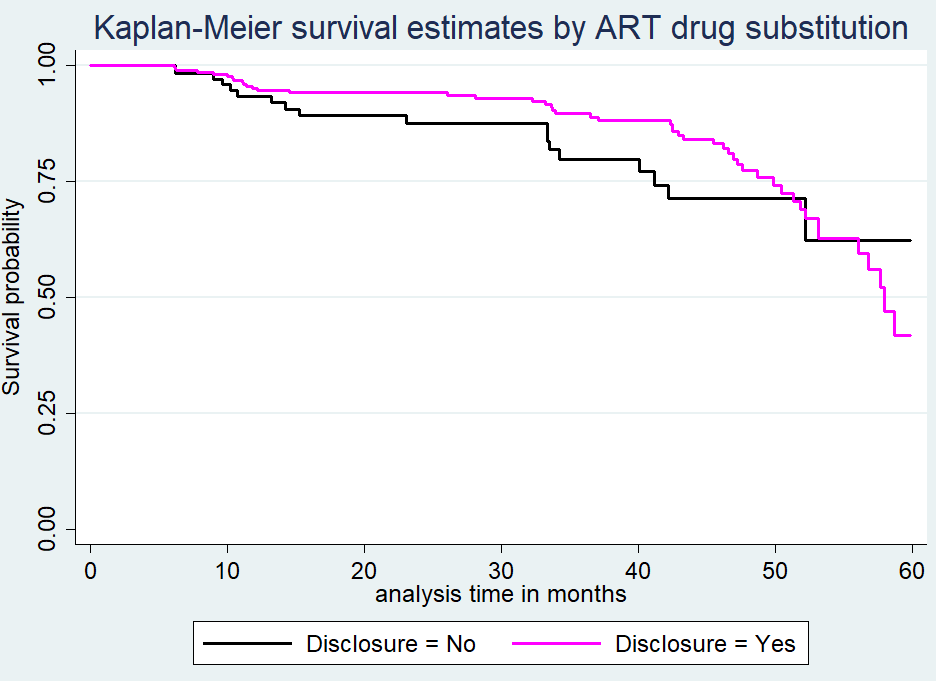

Supplement: S1 File — (DOCX) [file pone.0288132.s001.docx]
