# Supplementary material for: Incidence of switching to second-line antiretroviral therapy and its predictors among children on antiretroviral therapy at general hospitals, Northern Ethiopia: A survival analysis
Source: PLoS One. 2023 Sep 8;18(9):e0288132. doi: 10.1371/journal.pone.0288132 (PMC10490964; doi:10.1371/journal.pone.0288132)
Supplement: S2 File — (DOCX) [file pone.0288132.s002.docx]

**Supporting Table 1 (S1 Table).**

| Interval | Beg.  Total | Deaths | Lost | Survival | Std.  Error | [95% Conf. Int.] |
| --- | --- | --- | --- | --- | --- | --- |
| 0 - 12 | 424 | 18 | 123 | 0.9503 | 0.0114 | 0.9223 0.9684 |
| 12 - 24 | 283 | 6 | 59 | 0.9279 | 0.0144 | 0.8938 0.9513 |
| 24 - 36 | 218 | 11 | 40 | 0.8763 | 0.0203 | 0.8301 0.9106 |
| 36 - 48 | 167 | 16 | 82 | 0.7650 | 0.0315 | 0.6964 0.8202 |
| 48 - 60 | 69 | 14 | 55 | 0.5069 | 0.0599 | 0.3847 0.6169 |

**Supporting Table 1a**: Survival life table to assess second-line switching among HIV/AIDS infected children on first-line ART in public general hospitals, Northern Ethiopia, 2019/20, (n=424)

Note: The survival life-table for the five-year follow-up data was constructed by 12 months interval.

**Supporting Table 1b:** Cumulative failure life table to assess second-line switching among HIV/AIDS infected children on first-line ART in public general hospitals, Northern Ethiopia, 2019/20, (n=424)

| Interval | Beg.  Total | Deaths | Lost | Cum.  Failure | Std.  Error | [95% Conf. Int.] |
| --- | --- | --- | --- | --- | --- | --- |
| 0 - 12 | 424 | 18 | 123 | 0.0497 | 0.0114 | 0.0316 0.0777 |
| 12 - 24 | 283 | 6 | 59 | 0.0721 | 0.0144 | 0.0487 0.1062 |
| 24 - 36 | 218 | 11 | 40 | 0.1237 | 0.0203 | 0.0894 0.1699 |
| 36 - 48 | 167 | 16 | 82 | 0.2350 | 0.0315 | 0.1798 0.3036 |
| 48 - 60 | 69 | 14 | 55 | 0.4931 | 0.0599 | 0.3831 0.6153 |

**Supporting Table 2 (S2 Table):** Log-rank test for equality of survivor functions between groups to assess second-line switching among HIV/AIDS infected children on first-line ART in public general hospitals, Northern Ethiopia, 2019/20, (n=424)

|  | | **Log rank** | |
| --- | --- | --- | --- |
| N^o^ | **Variables** | **χ^2^** | **P > χ^2^** |
| 1 | Parent status | 12.87 | 0.0016* |
| 2 | WHO stage at baseline | 6.66 | 0.0098* |
| 3 | WHO stage at last visit | 18.33 | 0.0000* |
| 4 | Baseline CD4 count | 4.84 | 0.0489* |
| 5 | OI at baseline | 6.56 | 0.0105* |
| 6 | OI after initiation | 13.98 | 0.0002* |
| 7 | Anemia after initiation, | 19.81 | 0.0000* |
| 8 | Diarrhea after initiation | 2.50 | 0.1141* |
| 9 | Malnutrition at baseline | 1.96 | 0.1615* |
| 10 | Malnutrition after initiation | 4.57 | 0.0325* |
| 11 | TB at baseline | 23.99 | 0.0000* |
| 12 | TB after initiation | 2.91 | 0.0881* |
| 13 | Previous ART exposure | 1.59 | 0.0207* |
| 14 | Adherence to ART | 14.26 | 0.0002* |
| 15 | Drug toxicity | 38.91 | 0.0000* |

**Supporting Table 3 (S3 Table):** Test of proportional-hazards assumptions for each covariate

| Variables | Category | Parameters | | | |
| --- | --- | --- | --- | --- | --- |
|  |  | **Rho** | **chi^2^** | **Df** | **P-value** |
| Parent status | Both alive | Reference | Reference | Reference | Reference |
|  | Either died | 0.19860 | 2.74 | 1 | 0.0978 |
|  | Both died | -0.08299 | 0.46 | 1 | 0.4995 |
| OI at baseline | Yes | Reference | Reference | Reference | Reference |
|  | No | -0.01371 | 0.01 | 1 | 0.9083 |
| OI after ART initiation | Yes | Reference | Reference | Reference | Reference |
|  | No | -0.01353 | 0.02 | 1 | 0.8938 |
| Adherence to ART drugs | Optimal | Reference | Reference | Reference | Reference |
|  | Sub-optimal | 0.02704 | 0.06 | 1 | 0.8140 |
| Previous ART exposure | Yes | 0.01227 | 0.01 | 1 | 0.9259 |
|  | No | Reference | Reference | Reference | Reference |
| ART drug toxicity | Yes | -0.18289 | 2.32 | 1 | 0.1276 |
|  | No | Reference | Reference | Reference | Reference |
| Baseline WHO stage | Early-stage | Reference | Reference | Reference | Reference |
|  | Advanced stage | 0.03948 | 0.13 | 1 | 0.7135 |
| Latest WHO stage | Early-stage | Reference | Reference | Reference | Reference |
|  | Advanced stage | -0.02101 | 0.10 | 1 | 0.7494 |
| Baseline CD4 | Failed | -0.03802 | 0.22 | 1 | 0.6367 |
|  | Normal | 0.08071 | 0.41 | 1 | 0.5227 |
|  | Unknown | Reference | Reference | Reference | Reference |
| Anemia after ART start | No | Reference | Reference | Reference | Reference |
|  | Yes | -0.06782 | 1.01 | 1 | 0.3145 |
| Diarrhea after ART initiation | No | Reference | Reference | Reference | Reference |
|  | Yes | -0.14568 | 1.66 | 1 | 0.1982 |
| Malnutrition at baseline | No | Reference | Reference | Reference | Reference |
|  | Yes | 0.06452 | 0.30 | 1 | 0.5823 |
| Malnutrition after ART start | No | Reference | Reference | Reference | Reference |
|  | Yes | -0.02025 | 0.03 | 1 | 0.8725 |
| TB at baseline | No | Reference | Reference | Reference | Reference |
|  | Yes | 0.10654 | 0.67 | 1 | 0.4144 |
| TB after ART initiation | No | Reference | Reference | Reference | Reference |
|  | Yes | 0.00713 | 0.01 | 1 | 0.9434 |
| Follow up duration | | -0.06026 | 0.31 | 1 | 0.5801 |
| Global test | |  | 21.48 | 18 | 0.2599 |
