# Supplementary material for: Incidence of switching to second-line antiretroviral therapy and its predictors among children on antiretroviral therapy at general hospitals, Northern Ethiopia: A survival analysis
Source: PLoS One. 2023 Sep 8;18(9):e0288132. doi: 10.1371/journal.pone.0288132 (PMC10490964; doi:10.1371/journal.pone.0288132)
Supplement: Attachment — Submitted filename: Response to reviewers.DOCX [file pone.0288132.s005.DOCX]

**Manuscript title: Incidence of switching to second-line antiretroviral therapy and its predictors among children on antiretroviral therapy** **at general hospitals, Northern Ethiopia: A survival analysis (**PONE-D-22-19688**)**

Thank you for the opportunity to submit a revised version of our manuscript for your consideration. We appreciate the thoughtful and constructive comments of the editors and reviewers and have revised our manuscript accordingly. Detailed responses to each of the comments or que stions raised by the editors and reviewers appear below. We have indicated the text changes that have been made and the line number on the tracked changes manuscript at which our changes can be found.

**Editorial comments**

1. **Comment:** “Please ensure that your manuscript meets PLOS ONE's style requirements, including those for file naming."

**Author’s response:** We have reviewed the journal styles and requirements and adjust the files accordingly.

**Reviewer #1 Comments:**

1. **In the Introduction section:**

**Comment#1:** “The introduction contains great information but long, and would benefit from being condensed and streamlined.”

**Author’s responses:** We have tried to abridge the introduction part as much as possible (Please see Line 53-94).

2. **In the Methods and results:** Results have been presented very thoroughly but I recommend the following;

**Comment #1:** “Table 5: should be re-done to also show child-months, switch rate per 100 child-months. The crude estimates should be excluded and rather focus on the adjusted estimates”

**Author’s responses:** That is great! We acknowledge it was possible to report switch rate per 100 child-years or 1000 child-months. However, we preferred to report switch rate per 1000 child-months for discussion to make direct comparison with previous studies conducted abroad, which reported incidence rate per 1000 child-months. Regarding, the Table 5, we appreciate that the table was condensed and need to be simplified. As such, we removed the p-values column for both bi-variable and multivariable outputs since confidence intervals can be used to show significantly associated predictors (Kindly refer to Table 5). On the other hand, the reviewer’s suggestion to remove the crude HR is considerable. However, we prefer to keep it as it could not impose any negative impact on the table. Rather, including crude hazard ratios (CHR) will give better clarity for readers on which variables were transferred to multivariable analysis.

**Comment #2:** “Figures Kaplan Meier curves should be plotted for all the significant factors in the cox proportional hazards model and not just the failure estimates. (Stratified by for example WHO stage, being orphan, drug toxicity etc.)”

**Author’s response:** Thank you so much. We presented the overall KM failure estimate not to make the manuscript bulky and exhaustive for readers. Due to this reason, we have computed the KM curves for each independent variable and provided separately as supplementary files (Please refer to “Annex I”).

3. **Discussion and conclusion**:

**Comment #1:** “For the sentence ‘In this study, the median survival time was 58.7 months which is longer than the findings of previous studies; 35 months from a global pooled estimate by CIPHER, and 30 months in Europe and Thailand. The possible explanation could be the advancements in diagnostic and therapeutic measures. These include more frequent visits, increased access to viral load, and availability of more potent drugs nowadays than in the past.’ The author in his introduction alluded to the fact there were no studies conducted to evaluate factors tat predict switching to second-line ART regimens among children throughout the country so how does he justifies his possible explanations?”
**Author’s responses:** That’s right! We acknowledge that we have stated there were no studies conducted to evaluate factors that predict switching to second-line ART regimens among children throughout the country, Ethiopia (keep in mind that it does not mean no study globally). Nevertheless, those studies we used for comparison in the discussion section (both the CIPHER and Europe and Thailand studies) were conducted abroad in the Western countries. Since there are no similar studies in Ethiopia, where our study was conducted, we used the abovementioned studies for comparison purpose to discuss our findings with globally available evidence.

**Comment #2:** “Preferably remove actual results in the discussion and rather discuss key finding”

**Author’s responses:** Thanks again! We admit the comment and removed some details accordingly (Please see Line 266-271). We tried to focus on discussing main findings based on our study objectives.
**Comment #3:** “Discussion does not cover limitations of the study, there are several limitations associated with observational studies and should be considered within these findings”

**Author’s responses:** We admit the comment and addressed some limitations of the study at the end of “Discussion” section (Kindly see Line 332-340).

**Reviewer #2: Reflections**

**Comment #1:** “There are studies which addresses similar title in Ethiopia. What makes your different and added a new knowledge. There are also systematic review and meta-analysis studies.”

**Author’s responses:** As specified by the reviewer, there are several studies conducted in Ethiopia to address incidence of switch and ART treatment failure. However, those all available studies including the systematic reviews considered adult patients rather than the pediatric age group. We cannot generalize adult findings for the pediatric population due to the fact that the pediatric population is unique to adults requiring different guidelines, approach and drug formulations from adults. That is why the WHO endorsed a separate guidelines and drug formulations for children below the age of 15 years. Thus, as already described under the introduction part (Please refer to Line 93-95), we couldn’t find a single study conducted to address incidence of ART switch and its predictors among children (NB: not adults) in Ethiopia.

**Comment #2:** “Abstract, method part: sampling technique and P-value for declaration of Statically significance were not reported”

**Author’s responses:** We the authors accepted the comment and made the suggested changes succinctly to the manuscript file (Page 2, Line 31-38). Other detailed descriptions regarding the sampling technique and procedure were presented in the methods part of the manuscript file as the abstract should be short and succinct (Line 112-119).

**Comment #3:** “The statistical analysis suffers from the Table 2 fallacy, whereby multiple inferences are made using a single model. Moreover, it is very weird (actually wrong) that for all you used categorical predictors is

1. WFA at baseline, and
2. HFA at baseline because it dependents on age and the classification also varied for children less than five years and above five years also
3. You also missed MUAC being either <11.5 .
4. Also CD4 count at baseline has its classification based on WHO definition
Therefore, this manuscript has both stastical fallacy and predictors weird”

**Author’s responses:** We appreciate the issue raised by the reviewer, which is a core point. Regarding WFA and HFA, age and sex-appropriate growth charts should be used to get specific percentiles and z-scores for each child during data collection. This is because girls and boys have different growth charts because they grow in different patterns and at different rates. The same is true for each age category. However, after acquiring specific WFA and HFA values for each sample, the classification is consistent for both sexes and all age categories (please refer to WHO child growth standards, 2007). Therefore, interpretation of growth charts (classification) did not vary by age and sex since the variation in growth pattern and rate are fixed by using the recommended WHO growth charts for each individual participant.

The other point raised by the respected reviewer was about MUAC. Initially, we have included MUAC as one independent variable to be studied. Nevertheless, we couldn’t found such records in the patient charts. Hence, we have excluded MUAC since it was not documented on patient charts as the study was conducted retrospectively.

Regarding the CD4 count, formerly, the WHO and CDC recommend using CD4 percentage up to 5years of age and CD4 count afterward [kindly refer to WHO, 2016; CDC, 2014 (updated in 2022)]. However, recent pediatric HIV/AIDS guidelines showed that CD4 percentage provided little or no additional prognostic value compared with CD4 cell count regarding short-term disease progression in children aged <5years, and in older children as well (refer to AIDSinfo HHS, 2018). That’s why we used absolute CD4 count for all age categories. As already described by the reviewer, it’s true that CDC provided different CD4 count classifications based on age (<1year, 1-5years & >5years), with each age group has 3 or more categories. For this reason, since the variable is pertinent variable that its effect need to be assessed, we re-categorized this variable in to dichotomous values for analysis. While doing this, we consider the age variation and that is why we preferred to use CD4<200c/mm3 and >200c/mm3, which indicates severe immune suppression in all age groups according to “the Ethiopian national guidelines for comprehensive HIV prevention, care and treatment” (quoted from FMOH, 2017; pp 78). This is because, the CD4 category for infant could not be applicable for those above 1 year of age, whereas CD4 count <200c/mm3 could not be normal for all age groups such that it can be applicable for all age categories. Thus, we did this classification based on the national HIV/AIDS guideline. Thanks in advance.

**Comment #4:** “What is you reference to code VL at initiation as below or above 100”

**Author’s responses:** Well, actually we have classified viral load as below 1000 and above 1000copies/ml (**NB:** not below and above 100). Our references for this classification were Consolidated guidelines on the use of antiretroviral drugs for treating and preventing HIV infection: recommendations for a public health approach by WHO and the Ethiopian national guidelines for comprehensive HIV prevention, care and treatment by FMOH (clearly quoted in WHO, 2016 pp.131; FMOH, 2017 pp.81).

**Comment #5:** “Where is your Model fitness test if you used cox regression unless there is no indication in graphically or statically?”

**Author’s responses:** As already described in the manuscript file, the Cox proportional model fitness test was checked using Cox-Snell’s Residual test (graphically) in addition to the Schoenfield residual (global test) statistical assumption test (Kindly refer to Lines 149-158). The Cox Snell residual graph was included in the figure file labeled as “Figure 3” (Figure 3).

**Comment #6: “**Method part: You said about data collection periods April 1-26 /2019 and However I have assessed on Table 1: as you said it was in 2020GC it seems miss-behave on scientific environment and the data collection was even I have accepted it how it it is long duration influence the representativeness of your findings”

**Author’s responses:** Yes, it’s true that the data collection (extraction) was completed in the specified period. If it cause such ambiguity on readers, we can modify table captions to “2019/20” to include the data collection period (Please see Table 1-5). Nonetheless, as every researcher can understand, there are numerous tasks expected to be accomplished to complete the paper write-up starting from data cleaning, organization, coding, entry, data management, analysis and report writing. The data collection was conducted in 2019 and manuscript write-up was completed around the beginning of 2020. Hence, the 2020G.C in the table captions and figure legends implies the time of manuscript write-up, but not the data collection period. This could not be considered as a fallacy since both contents were described briefly in their respective places. Regarding the representativeness issue, firstly, we have used a five-year follow-up data and it is not a cross-sectional study (snapshot data). Therefore, it is not expected to be influenced with a few months lapsed. Secondly, such issues would be more applicable if the study was something affected by seasonal changes (for instance studies on asthma and allergies) as well as disasters, which was not the case in ART service delivery system. So, we can assure that representativeness issue will not be a worry in this study.

**Comment #7: “**Model you used seems to me inappropriate as you used , please it is fact than treatment switching can be caused by more than one factors and hazard could not be constant , there for the brain of the research is missed and it is more efficient that predictor should to be identified by Computing risk regression rather than cox regression , or distribution of treatment fail in causes weird distribution both intrinsic and proximal risk factors , there for it also appropriated by Exponential regression is more also properties , there for cumbersome reading more than is because failed data management”

**Author’s responses:** Well, nice point was raised here and we appreciate for the concern paid. It’s true that treatment switching can be caused by several factors. To begin with, competing risks regression is a different concept, and actually not related to the number of independent /causative factors. Competing risk regression models should be considered when more than one events of interest (failures) exist in the study, and if these events are dependent and compete each other. In such cases, applying the Kaplan-Meier estimates and standard survival analysis methods such as Cox proportional hazard regression will provide biased outputs. Because, this method will assume just a single event and consider all the other outcomes as censor (Haesook T. Kim, 2007; Tai BC et al., 2011, James J d et al., 2013). However, in our study, we have a single definite event (switching to second-line ART), and no other competing events exist. Consequently, Cox proportional hazard model was an appropriate model to analyze our data, and competing risk regression could not be an option in this case. The other point raised by the reviewer was “hazard could not be constant”. In Cox proportional hazard regression, the hazard ratios for a certain event remains constant over time. This is one of the main assumptions of Cox PH regression model (Longjian Liu, 2018). Thus, this should not be a worry anymore. Thanks again!

**Reviewer #3 Reflections:**

1. **General comments**

**Comment #1: “**But the manuscript is with a lot of grammatical and spelling errors and thus needs to be thoroughly revised by a native English speaker.”

**Author’s responses:** We tried to exhaustively revise the whole manuscript for grammar and typographic suitability.

**Comment #2: “**Both in the abstract and the full text, abbreviations should be written entirely in their first appearance. Then after, it could be better to use the abbreviation throughout the whole text.”

**Author’s responses:** Use of abbreviations moderated accordingly throughout the text.

**Comment #3: “**The authors should be in line with the journal submission guidelines.”

**Author’s responses:** We tried to re-check the PlosOne author guidelines for journal requirements and tried to follow these requirements as much as possible.

2. **On the Topic:**

**Comment #1:** “Better to revise the topic as, Incidence of switching to second-line therapy and its predictors among children on antiretroviral therapy at general hospitals, Northern Ethiopia: A survival analysis.”

**Author’s responses:** Thank you so much! We think the reviewer’s suggestion is more direct forward and, thus, it was modified as directed (Title page, Line 2).

3. **In the ABSTRACT part:**

**Comment #1: “**This section should incorporate all the main methods you used in conducting the research. So, you have to add the sample size, the population of the study, the study period, and the sampling procedure to recruit study participants.”

**Author’s responses:** We tried to make it succinct not to exceed the word limit. Now, we accepted the comment and have included the abovementioned points accordingly (please see Lines 31-38).

**Comment #2:** “Page 2, lines 34-35: The authors stated that “…… log-rank test, and life table were used for data description….”; but there is no any life-table and log-rank value in the result section of the main document. So, better to revise this.”

**Author’s responses:** We have used log-rank test to compare survival curves for the presence of difference in the incidence of survival among the groups and life-table was constructed to estimate probabilities of failure at different time intervals. However, we have just reported pertinent findings since it could be exhaustive for readers to present every finding in the manuscript. We described these parameters here just to show we have conducted all the necessary steps of analysis. If it is necessary, the life table and log-rank test output were supplied separately as “supplementary file” (Please check Annex II).

**Comment #3: “**How did you check the model adequacy for your data?”

**Author’s responses:** Model adequacy is all about whether the model assumptions are fulfilled and the data is fitted to the selected model. Accordingly, we have checked the model assumptions statistically using Schoenfield residual (global) test and model fitness was checked graphically by Cox Snell’s residual. The corresponding findings for both tests showed that the data was fitted well to the selected model, and were documented in the main manuscript (Kindly refer to Line 149-158). It will not be appropriate to present every detail in the abstract section.

**Comment #4: “**Be in line with the authors’ submission guideline.”

**Author’s responses:** We tried to fulfill the journal requirements and tried to follow these author guidelines as much as possible. Some abbreviations such as SPSS, AOR, AHR, OR, & HR are considered as standard statistical abbreviations in routine scholars, thus, can be used in the abstract section not to make it bulky. Journal guidelines also put as optional but not mandatory requirements in such scenarios.

**Comment #5: “**Page 2, Line 42: “…… advanced latest WHO stage…” use either ‘advanced WHO stage’ or ‘latest WHO stage’.”

**Author’s responses:** Thank you! But, the two terms have independent implication, in which “advanced” indicates the WHO clinical stage whereas “latest” refers to the timing when the data was collected (i.e. WHO stage at the end of study follow-up). Be reminded that we have baseline data (taken at the beginning of study follow-up) and latest/recent data (taken at the end of study follow-up).

**Comment #6: “**Page 2, lines 47-48: “Further research is also needed to evaluate the effect of drug resistance.” At the abstract section, please recommend based on your main findings. The recommendation under the quotation should be included in the main document.”

**Author’s responses:** We admit the comment and included this statement under the last paragraph of the “Discussion” section as a limitation (Kindly see Lines 332-340).

4. **In the Introduction section:**

**Comment #1: “**Since the introduction lacks idea flows, this section requires grammatical editing and rearrangements of sentences and paragraphs.”

**Author’s responses:** We followed the global to local principle, and we tried to make some arrangements as required. We have revised for typographic issues as well (Lines 53-94).

**Comment #2:** “Page 3, lines 57 – 62: “Switching to second-line ART was defined as changing ≥2 new drugs, ……. with the reason of switch documented as treatment failure”. I recommend the authors remove this definition from the introduction and incorporate it into the operational definition of the method section.”

**Author’s responses:** We agreed with the reviewer and moderated as directed (please check on Line 121-125).

**Comment #3:** “ ‘Page 4, lines 85 – 86: “Shreds of evidence also suggested that the costs of second-line ART drugs were more than double to triple compared with first-line regimen costs’. What is the implication of this sentence in accordance with your objective? I recommend the author remove it.”

**Author’s responses:** Actually, this statement was to emphasis the effect of increased incidence of second-line switching on ART program costs. Meaning that since second-line ART options are costly compared to first-line regimens, increased incidence of switching could drive-up the health cost. However, we have merged this idea and expressed in short phrases rather than completely removing it (please refer to Line 76-81).

**Comment #4: “**It is expected more from the author to show what other literature has reported regarding the incidence of switching to a second-line regimen.”

**Author’s responses:** We have provided all available but limited evidence after extensive literature search and already described in the main manuscript (Kindly look at Lines 58-59 for global data and Lines 87-90 for incidence in Ethiopia). As we have stated in the gap statement, unlike in the adults, there is paucity of data regarding second-line switching in the pediatric age group.

**Comment #5: “**Page 4, lines 91 – 92: “Ethiopia is one of the high HIV-burden countries and does not have appropriate ART drug formulations for children beyond the 2nd line.” How could the lack of ART drug formulations beyond the 2nd line be related to a delay in switching to 2nd-line ART?”

**Author’s responses:** Thanks again! This statement is to show how much delay in second-line switching will affect the ART service, especially in Ethiopia. As we have described in the main manuscript, prolonged treatment with a failed first-line regimen and delay in switching could result in a 46% raised chance of failure to second-line therapy (please see page 3, Line 74-76). Therefore, children who switched after significant delay will be at an increased risk of failure to second-line regimens as well. Afterward, if there is no appropriate pediatric drug formulations, children will have no option and just have to wait for death to come soon. Hence, this statement is important to describe the effect of switching delayance in such countries. As such, we prefer to keep it as it is.

**Comment #6: “**It is not recommended to use unusual words in research writing, such as Albeit.”

**Author’s responses:** We thought it should not be a concern as far as it is formal language, but the word “albeit” was replaced with “despite” anyways (Line 91). Respect!

5. **In the METHODS AND MATERIALS**

**Comment #1: “**You missed a sub-heading “study setting”.

**Author’s responses:** Well acknowledged! We have incorporated the sub-heading “study settings and population” in the suggested place (Line 96).

**Comment #2: “**Be consistent in using phrases like “HIV/AIDS-infected children” vs “HIV/AIDS patient” throughout the document.”

**Author’s responses:** Great! We find and replaced all with “HIV/AIDS-infected children” throughout the text.

**Comment #3: “**When was the data collected? Why didn't you incorporate children who started ART after 2018?”

**Author’s responses:** As it was already described in the manuscript (see Line 141), the data collection/data extraction was conducted in April 2019 by reviewing a five years data from recordings of children receiving first-line ART from January 2014 to December 2018 at the selected hospitals (Line 103). This is because, there should be a minimum of three months follow-up data to declare treatment outcome such as switching and treatment failure (one of the reasons for switching). Before three months follow-up, patients will be followed for initial treatment responses (like adherence, drug tolerance, IRIS, toxicity), which probably could lead to the outcome rather than the event of interest (the outcome) itself (FMOH, 2017). Due to this reason, we included children who started ART follow-up until the end of 2018, but excluded after then.

**Comment #4: “**What type of cohort that the authors used? Was it open or closed cohort?”

**Author’s responses:** Children who started receiving first-line ART between 1^st^ January, 2014 and 31^st^ December, 2018 were incorporated to the study. Some children might be on follow-up for the whole 60 months and some others might exit anytime in between due to any reason (either as event of censor). Whereas, some of them might be included in 2015, 2016, 2017, or 2018, and anytime in between. So it can be considered as open type. We hope the study population statement can give a clue for such confusions (Line 102-104).

**Comment #5: “**What does a design effect of 1.5 mean? Is there a fractional design effect?”

**Author’s responses:** Of course yes! Design effect can be any figure mostly between 1 and 3, and in some cases can be extended from 10 to 30 depending on the variation in population characteristics and the sampling technique applied (UNICEF multiple indicator cluster survey manual, 2011; Alimohamadi Y et al., 2019; Gene Shackman, 2001). So, in our case, we have applied design effect because of the sampling technique we used although there is no sufficient evidence showing differences in the population characteristics from the ART context. We preferred to use 1.5 because we did not use complicated sampling steps, and the use of similar ART guideline with equivalently trained professionals will reduce the difference in ART data among the population in study.

**Comment #6: “**Was there any ground or reason to select three from five hospitals? Why not two of four?”

**Author’s responses:** Well, we have randomly selected three hospitals among five hospitals that were ready for selection. We did not consider two hospitals to achieve better population representativeness, and not four or five for cost-effectiveness. As we all know, sampling is required to minimize unnecessary spending and extra efforts required for data collection and data handling. Therefore, as far as we used appropriate methodological and sampling steps to collect accurate data, which is representative of the target population, it could not be a problem to use samples rather than census.

**Comment #7: “**Page 5, lines 125 – 126: remove the description “The detail of the sampling and participant selection procedure was presented in the flowchart below”. Only (Figure 1) could be enough.”

**Author’s responses:** Comment admitted and moderated accordingly (Line 119).

**Comment #8: “**Figure 1: The sum of the bracketed numbers (66 and 8) is 74, not 72.”

**Author’s responses:** Thank you! That was edition error and corrected in the figure files (Figure 1).

**Comment #9: “**Why did the authors exclude those eight patients who didn't have follow-up data after ART initiation? Why didn’t you consider these patients as a censored?”

**Author’s responses:** That is a brilliant idea. This is because, in incidence studies, all the participants must be at risk to develop the event of interest (switching in our case) initially. There should be a minimum of three months follow-up data to declare treatment outcome such as switching and treatment failure (one of the reasons for switching). This means, switching could not be diagnosed on a patient who had been followed for 1 week or 1 month. Incorporating this patient could be like finding a milk from an ox. Censored cases should be at risk initially with a possibility of being diagnosed with the event, but did not develop until the end of follow-up yet. Before three months follow-up, patients will be followed for initial treatment responses (like adherence, drug tolerance, IRIS, toxicity), which probably could lead to the outcome rather than the event of interest (the outcome) itself (FMOH, 2017). Due to this reason, we excluded those children who had not been on ART follow-up for at least three months.

**Comment #10: “**If 430 patients fulfilled the inclusion criteria, why have you only analyzed 424 patients? Why didn’t you survey all of them?”

**Author’s responses:** That was possible actually. Initially, we didn’t think in such a way. We thought it could not be reasonable to consider a survey after specifying random samples with predetermined sample size. However, for the current situation, we strongly believe that the data we used could not be influenced by exclusion of six cases as far as the scientifically estimated sample size was attained. In fact, that was a great gain for the future encounters and thanks allot!

**Comment #11: “**Censored (Lines 131-134): Is there a distinction between 'those who did not switch to the second-line regimen during follow-up' and 'those who were still on first-line at the end of follow-up'?”

**Author’s responses:** Yes, these are two different concepts. **“**Those who did not switch to the second-line regimen during follow-up” is a general introductory phrase to infer all censored cases (including lost, transferred out, died, exceed 15^th^ birthday during follow-up, and on first-line at the end of follow-up). In this case, censor can be recorded at any time during follow-up (either lost immediately after entry, died at mid-term, or can be at the completion of follow-up). However, 'those who were still on first-line at the end of follow-up' are just one component of the censored group. In this condition, censor is recorded just only if the child remain on first-line ART at the completion of study follow-up period.

**Comment #12: “**Also, the description “Children were at risk of switching from ART start until the earliest switch or censor” couldn’t be an operational definition. So, please remove it.”

**Author’s responses:** Comment admitted and requested changes made accordingly (Line 121-129).

**Comment #13: “**Adherence (Lines 135-137): when did you say the patient’s level of adherence was either Good, Fair, or Poor? This should be revised.”

**Author’s responses:** The requested details were provided in the main document (Line 132-134).

**Comment #14: “**Clearly show the statistical model you used?”

**Author’s responses:** The required analysis steps and regression model were described briefly in the manuscript file (Kindly look in Lines 149-158).

**Comment #15: “**Why did you compute Harrell’s C statistics? You have to clearly put in the document its role.”

**Author’s responses:** Harrell’s C test is one of the Cox regression assumption tests, which is conducted to determine the concordance probability. The necessary details of Harrell’s C test to our data are incorporated in the main document (please check in Lines 153-155).

**Comment #16: “**Line 149-150: “Life-table was used to estimate the ……”; but there is no any life table in the document.”

**Author’s responses:** Thank you! We have constructed and provided the life-table as supplementary file (Annex II, supplementary file 1a and 1b).

**Comment #17: “**Lines 152 – 153: “Those variables having P≤0.20 in the bi-variable analysis …” Vs “variables ≤0.20 in the univariate analysis (in the abstract section)”: make it consistent.”

**Author’s responses:** Changes made as directed (Line 37).

**Comment #18: “**Lines 153 – 154: “Proportional hazard model assumptions were checked using the Schoenfield residual test (p=0.26)”. Proportional hazard assumption is conducted for each exposure variable; that means you couldn’t put a common p-value for different variable. So, a single Schoenfield residual value of 0.26 should be of the overall fitness, not proportional hazard assumption for variables.”

**Author’s responses:** We describe that the PH assumption was checked using global test (Schoenfield residual test), and put the overall output for simplicity. It could be illogical to present every finding in the manuscript file. So, if it is relevant, we have supplied the Schoenfield residual test output for each covariate separately as supplementary file (Annex II, supplementary file 3).

**Comment #19: “**Where did pretest was conducted? Why?”

**Author’s responses:** The pretest was conducted at Ayder Comprehensive Specialized Hospital in order to check clarity and consistency of the tool so that unclear and confusing items were modified as needed (Lines 163-165). Since the study was conducted retrospectively via review of records, the issue of participant sensitization and the like could not be the concern in this case. In this sense, site selection was not that much challenging as far as the pretest was conducted in the ART records. So, pretest was held at Ayder Comprehensive Specialized Hospital.

**Comment #20: ‘**Lines 163-164: Since it is already stated in the eligibility criteria above, it is better to remove the description “Individual records with incomplete data were excluded from the analysis”.’

**Author’s responses:** Comment accepted and changes were made as recommended (Line 163-168).

6. **In the RESULTS section:**

**Comment #1: “**Over all, the result section is full of grammatical errors and needs a thorough revision.”

**Author’s responses:** We tried to revise the whole manuscript for grammar and typographic issues.

**Comment #2: “**Medians should be described along with their interquartile range (IQR).”

**Author’s responses:** Amended as directed (Line 182-185).

**Comment #3: “**Page 7, Lines 178 – 180: the authors reported that “two hundred eleven (49.8%) children were males, of which 16.6% fulfilled the switch criteria”. What exactly does it mean by "fulfilled the switch criteria"?”

**Author’s responses:** This was to mean fulfilling the definition of second-line switching. As already clearly described under the operational definition sub-heading, there are three criteria to define switching (Line 121-125). Of those, at list one criteria must be documented to declare second-line switching. But, we have rephrased the statement to avoid confusion for readers (Line 183-186).

**Comment #4: “**Line 189 - 191: “…. had less than 475cells/mm3” what type of cells? Please specify clearly in the document.”

**Author’s responses:** Appreciated and required changes made (Line 195).

**Comment #5: “**…. advanced WHO clinical stage 25 (22.5%) were”, what to mean?”

**Author’s responses:** Thank you again! The statement was rephrased in clearer terms (Lines 196-198).

**Comment #6: “**Figure 2: why you prefer failure cure other than survival curve?”

**Author’s responses:** Both the survival and the failure curves can be used. However, we prefer the failure curve just because it was suitable for direct interpretation. Since the primary outcome interest (event) was second-line switch (failure), using the survival curve could require an indirect interpretation for the failure.

**Comment #7: “**Lines 225 – 226: “...... and it crosses the survival function at a survival probability of 0.5”; so, what it indicates?”

**Author’s responses:** Great, that was a statement with an incomplete idea. We made necessary changes in the main document (Line 238-240).

**Comment #8: “**You repeatedly wrote the sentence “Variables with a p-value of ≤0.20 in the univariate analysis were transferred to the multivariable analysis after checking the model fitness and proportional assumptions.” So, please remove it from here.”

**Author’s responses:** Statement removed as suggested (Lines 247-252).

**Comment #9: “**Table 5: I recommend to the authors to remove the columns with P-values because CI are enough to show that the variable was significantly associated.”

**Author’s responses:** We agreed with the respected reviewer’s idea and we made changes accordingly (Kindly refer to Table 5).

**Comment #10: “**The authors should interpret and write in text the common and significantly associated variables.”

**Author’s responses:** We have presented only the significantly associated variables both in the results and the discussion sections.

**Comment #11: “**If the authors computed a Cox-Snell residual test (as stated in the method section), where is the graph?”

**Author’s responses:** We have provided the graph in the “figure files” (Figure 3).

7. **On the Discussion part:**

**Comment #1: “**Page 13, lines 248 – 253: since this paragraph has already been stated, there is no need to repeat it.”

**Author’s responses:** We appreciate the idea! By principle, the first paragraph of discussion section should begin by highlighting the pertinent findings so that readers will have a clue on the main ideas to be discussed. Hence, with due respect, we prefer to keep this one as presented.

**Comment #2: “**Lines 254 – 256: Is the cumulative incidence expressed as a rate or as a proportion?”

**Author’s responses:** Cumulative incidence (the proportion of people who develop the outcome during a fixed block of time) is expressed in proportion whereas incidence rate (a true rate where the denominator is the total of the group's individual times at risk) should be expressed in rate. Despite we calculated incidence rate, we couldn’t found previous studies, which reported second-line switch incidence rate. Thus, we used incidence proportion to compare our finding with the existing literature here in the discussion section.

**Comment #3: “**Over all, the discussion section needs a thorough revision.”

**Author’s responses:** We gone through the whole document for write-up and possible unclear statements.

**Comment #4: “**It would be better to add a limitation to this study.”

**Author’s responses:** We have addressed limitations under the last paragraph of the discussion section (Please check in Lines 332-340).
