# Supplementary material for: Incidence of switching to second-line antiretroviral therapy and its predictors among children on antiretroviral therapy at general hospitals, Northern Ethiopia: A survival analysis
Source: PLoS One. 2023 Sep 8;18(9):e0288132. doi: 10.1371/journal.pone.0288132 (PMC10490964; doi:10.1371/journal.pone.0288132)
Supplement: Attachment — Submitted filename: Reviewer comments.docx [file pone.0288132.s004.docx]

**GENERAL COMMENTS**

- First, I would like to appreciate the authors’ intention towards identifying predictors of switching to second-line antiretroviral therapy, which is a devastating condition globally. It gives a clue about what to do in order to reduce treatment failure.
- But the manuscript is with a lot of grammatical and spelling errors and thus needs to be thoroughly revised by a native English speaker.
- Both in the abstract and the full text, abbreviations should be written entirely in their first appearance. Then after, it could be better to use the abbreviation throughout the whole text.
- The authors should be in line with the journal submission guidelines.
- Kindly find below my specific comments to help improve the quality of the manuscript:

**Topic**: Better to revise the topic as, Incidence of switching to second-line therapy and its predictors among children on antiretroviral therapy at general hospitals, Northern Ethiopia: A survival analysis.

**ABSTRACT**:

Methods:

- This section should incorporate all the main methods you used in conducting the research. So, you have to add the sample size, the population of the study, the study period, and the sampling procedure to recruit study participants.
- Page 2, lines 34-35: The authors stated that “…… log-rank test, and life table were used for data description….”; but there is no any life-table and log-rank value in the result section of the main document. So, better to revise this.
- How did you check the model adequacy for your data?

**Results and conclusion:**

- Be in line with the authors’ submission guideline.
- Page 2, Line 42: “…… advanced latest WHO stage…” use either ‘advanced WHO stage’ or ‘latest WHO stage’.
- Page 2, lines 47-48: *“Further research is also needed to evaluate the effect of drug resistance.”* At the abstract section, please recommend based on your main findings. The recommendation under the quotation should be included in the main document.

**INTRODUCTION:**

- Since the introduction lacks idea flows, this section requires grammatical editing and rearrangements of sentences and paragraphs.
- Page 3, lines 57 – 62: *“Switching to second-line ART was defined as changing ≥2 new drugs, ……. with the reason of switch documented as treatment failure”.* I recommend the authors remove this definition from the introduction and incorporate it into the operational definition of the method section.
- Page 4, lines 85 – 86: *“Shreds of evidence also suggested that the costs of second-line ART drugs were more than double to triple compared with first-line regimen costs”.*
  - What is the implication of this sentence in accordance with your objective? I recommend the author remove it.
- It is expected more from the author to show what other literature has reported regarding the incidence of switching to a second-line regimen.
- Page 4, lines 91 – 92: “*Ethiopia is one of the high HIV-burden countries and does not have appropriate ART drug formulations for children beyond the 2nd line”*
  - How could the lack of ART drug formulations beyond the 2nd line be related to a delay in switching to 2nd-line ART?
- It is not recommended to use unusual words in research writing, such as Albeit.

**METHODS AND MATERIALS**

- You missed a sub-heading “study setting”.
- Be consistent in using phrases like “HIV/AIDS-infected children” vs “HIV/AIDS patient” throughout the document.
- When was the data collected? Why didn't you incorporate children who started ART after 2018?
- What type of cohort that the authors used? Was it open or closed cohort?

**Sample size determination and sampling procedure:**

- What does a design effect of 1.5 mean? Is there a fractional design effect?
- Was there any ground or reason to select three from five hospitals? Why not two of four?
- Page 5, lines 125 – 126: remove the description *“The detail of the sampling and participant selection procedure was presented in the flowchart below”.* Only (Figure 1) could be enough.
- **Figure 1:** The sum of the bracketed numbers (66 and 8) is 74, not 72.
  - Why did the authors exclude those eight patients who didn't have follow-up data after ART initiation? Why didn’t you consider these patients as a censored?
  - If 430 patients fulfilled the inclusion criteria, why have you only analyzed 424 patients? Why didn’t you survey all of them?

**Operational definitions:**

- **Censored (Lines 131-134):** Is there a distinction between 'those who did not switch to the second-line regimen during follow-up' and 'those who were still on first-line at the end of follow-up'?
  - Also, the description **“**Children were at risk of switching from ART start until the earliest switch or censor” couldn’t be an operational definition. So, please remove it.
- **Adherence** (Lines 135-137): when did you say the patient’s level of adherence was either Good, Fair, or Poor? This should be revised.

**Data processing and analysis:**

- Clearly show the statistical model you used?
- Why did you compute Harrell’s *C* statistics? You have to clearly put in the document its role.
- Line 149-150: *“Life-table was used to estimate the ……”;* but there is no any life table in the document.
- Lines 152 – 153: *“Those variables having P≤0.20 in the bi-variable analysis …”* Vs “variables ≤0.20 in the univariate analysis (in the abstract section)”: make it consistent.
- Lines 153 – 154: *“Proportional hazard model assumptions were checked using the Schoenfield residual test (p=0.26)”.*
  - Proportional hazard assumption is conducted for each exposure variable; that means you couldn’t put a common p-value for different variable.
  - So, a single Schoenfield residual value of 0.26 should be of the overall fitness, not proportional hazard assumption for variables.

**Data quality assurance:**

- Where did pretest was conducted? Why?
- Lines 163-164: Since it is already stated in the eligibility criteria above, it is better to remove the description *“Individual records with incomplete data were excluded from the analysis”.*

**RESULTS**:

- Over all, the result section is full of grammatical errors and needs a thorough revision.
- Medians should be described along with their interquartile range (IQR).
- Page 7, Lines 178 – 180: the authors reported that “two hundred eleven (49.8%) children were males, of which 16.6% fulfilled the switch criteria”. What exactly does it mean by "fulfilled the switch criteria"?
- Line 189 - 191: “…. had less than 475cells/mm3” what type of cells? Please specify clearly in the document.
  - “ …. advanced WHO clinical stage 25 (22.5%) were”, what to mean?
- **Figure 2:** why you prefer failure cure other than survival curve?
  - **Lines 225 – 226: *“......*** *and it crosses the survival function at a survival probability of 0.5****”;*** so, what it indicates?
- You repeatedly wrote the sentence ***“****Variables with a p-value of ≤0.20 in the univariate analysis were transferred to the multivariable analysis after checking the model fitness and proportional assumptions.****”*** So, please remove it from here.
- **Table 5:** I recommend to the authors to remove the columns with P-values because CI are enough to show that the variable was significantly associated.
  - The authors should interpret and write in text the common and significantly associated variables.
- If the authors computed a Cox-Snell residual test (as stated in the method section), where is the graph?

**DISCUSSION:**

- Page 13, lines 248 – 253: since this paragraph has already been stated, there is no need to repeat it.
- Lines 254 – 256: Is the cumulative incidence expressed as a rate or as a proportion?
- Over all, the discussion section needs a thorough revision.
- It would be better to add a limitation to this study.
